# Atypical hemolytic uremic syndrome and acute tubular necrosis induced by complement factor B gene (CFB) mutation: A case report

**DOI:** 10.1097/MD.0000000000025069

**Published:** 2021-03-19

**Authors:** Hao Wu, Sensen Su, Lin Li, Li Zhang

**Affiliations:** Department of Nephrology, The First Hospital of Jilin University, Changchun, Jilin, China.

**Keywords:** atypical hemolytic uremic syndrome, case report, complement, complement factor B, gene mutation

## Abstract

**Rationale:**

Atypical hemolytic uremic syndrome (aHUS) is an uncommon and serious disease that manifests hemolytic anemia, thrombocytopenia, and acute kidney injury. Genetic complement abnormalities have been shown to be responsible. Compared with the aHUS caused by other mutated genes, aHUS secondary to CFB mutation in adults is extremely rare. We report an adult with CFB mutation developing aHUS.

**Patient concerns:**

A 56-year-old man was admitted for 4-day history of nausea and fatigue, anuria for 2 days, and unconsciousness for 10 hours.

**Diagnoses:**

The patient presented with life-threatening anemia, thrombocytopenia, acute kidney injury, and nervous system abnormalities. The patient had schistocytes on the peripheral blood smear, increased lactate dehydrogenase (LDH), and plasma-free hemoglobin levels. The patient was later found to harbor a pathogenic variant in the CFB gene (C.1598A>G), and was diagnosed with aHUS and acute kidney injury.

**Intervention:**

The patient was treated by plasmapheresis, continuous renal replacement therapy, blood transfusion, and anti-infective and antihypertensive treatment.

**Outcomes:**

After the treatment, the patient's consciousness returned to normal, and the hemoglobin, platelet, and serum creatinine recovered. The disease activity remained quiescent during the follow-up.

**Lessons:**

A rare heterozygous variant c.1598A>G p.Lys 533Arg in the *CFB* gene, which was associated with adult-onset aHUS, was described and successfully treated. This case can help in understanding the early diagnosis and effective therapies of this rare disease.

## 1 Introduction

As a rare and serious microvascular thrombotic disorder, atypical hemolytic uremic syndrome (aHUS) is characterized by microangiopathic hemolytic anemia, platelet consumption, and the development of acute kidney injury.^[1]^ The aHUS is caused by the dysregulation of the alternative complement pathway,^[1,2]^ and aHUS patients have been found to have mutations that involve C3, complement factor H (CFH), factor I (CFI), factor B (CFB), and membrane cofactor protein (MCP, or CD46).^[2]^ Compared with other mutated genes, mutations that involve CFB are rare, while adult-onset cases are even rarer.^[3–6]^

We report an adult with life-threatening anemia, thrombocytopenia, nervous system abnormalities, and acute kidney injury, who harbored a pathogenic variant in the CFB gene (C.1598A>G). The patient was diagnosed with aHUS and acute tubular injury, and was successfully treated with plasmapheresis.

## 2 Case report

### 2.1 Clinical presentation

A 56-year-old male was admitted for 4-day history of nausea and fatigue. The patient also had anuria for 2 days, and was unconscious for 10 hours. Furthermore, the patient had no medically important history. On the physical examination, the patient was comatose, and had a pale appearance and impaired blood oxygenation. Rales and rhonchi were present in the bilateral lungs.

The laboratory investigations yielded features suggestive of microangiopathic hemolytic anemia, including a hemoglobin level of 35 g/L, schistocytes on the peripheral blood smear (1%), and increased lactate dehydrogenase (LDH) (416 IU/L) and plasma-free hemoglobin levels. The patients direct Coomb test results were negative, and the platelet and white blood cell count were 88 × 10^9^/L and 19.43 × 10^9^/L, respectively. Furthermore, the serum creatinine and blood urea nitrogen (BUN) levels were 1046 and 63.19 mmol/L, respectively, suggestive of renal impairment. The urinalysis revealed microscopic hematuria and 3+ proteinuria. There was no evidence of infection caused by hepatitis B, C virus, or human immunodeficiency virus. The anti-DNA and antiphospholipid antibody, and cryoglobulin were all negative. The chest computed tomography revealed pulmonary edema, and the culture revealed *Staphylococcus aureus*.

Immediately after admission, continuous renal replacement therapy (CRRT) and mechanical ventilation were started, and blood transfusion was administered. After 1 day of treatment, the patient regained consciousness with less dyspnea, and the investigators discontinued the mechanical ventilation. However, the patient's hemoglobin and platelet count remained persistently low, accompanied by anuria and hypertension.

**Figure 1:**
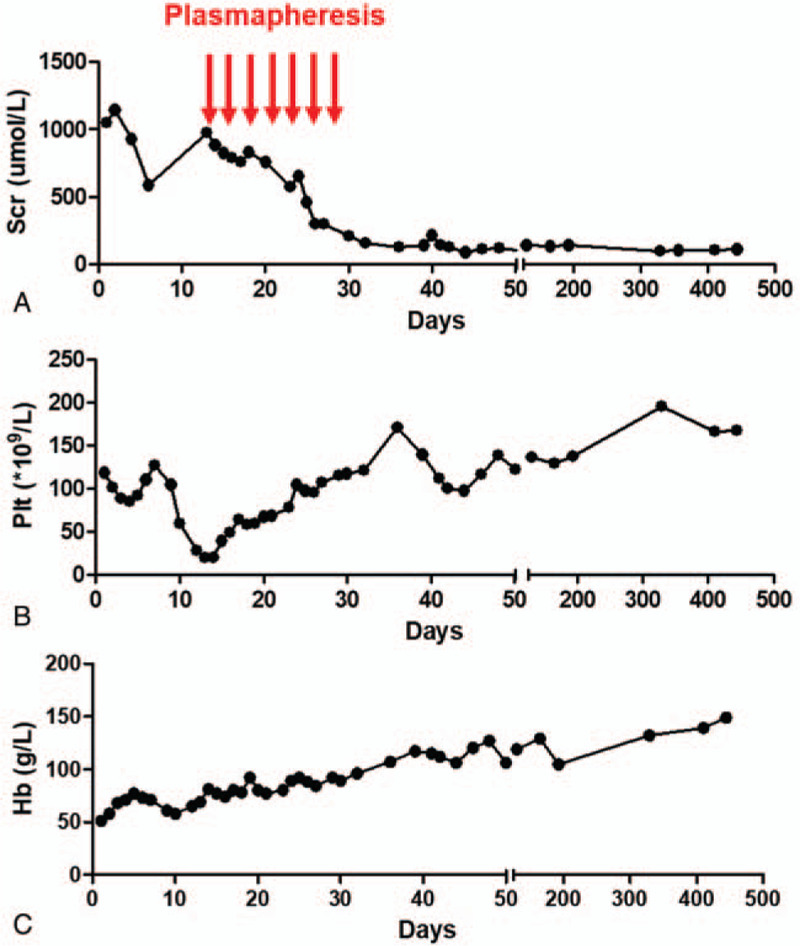
Biological parameters of the patient during the course of disease. (A) The changes of serum creatinine during the course of disease are shown. (B) The changes of the platelets during the course of disease are shown. (C) The changes of the hemoglobin during the course of disease are shown. The normal value of Scr multiply by 88.4 μmol/L, the value of hemoglobin multiply by 120 g/L, and the platelets from 100–300 × 10^9^/L. Hb = hemoglobin, PLT = platelet, Scr = serum creatinine.

On the basis of the fact that the patient had microangiopathic hemolytic anemia, thrombocytopenia, and acute kidney injury, the diagnosis of aHUS was considered. Plasmapheresis with a dose of 3000 mL per session for 7 sessions was initially prescribed (Fig. 1A). In addition, antibiotics were given, and the hemodialysis was continued. Losartan (200 mg/day) was given to reduce blood pressure for potential vascular protection. After 2 sessions of plasmapheresis, the patient's amount of urine gradually increased to the degree of polyuria, and this normalized thereafter. Meanwhile, the patient's serum creatinine decreased (Fig. 1A), while the platelet (Fig. 1B) and hemoglobin (Fig. 1C) recovered. After 15 months of follow-up, the patient's platelets and hemoglobin remained normal, and the serum creatinine trended toward normality (Fig. 1).

### 2.2 Pathology of renal biopsy

**Figure 2:**
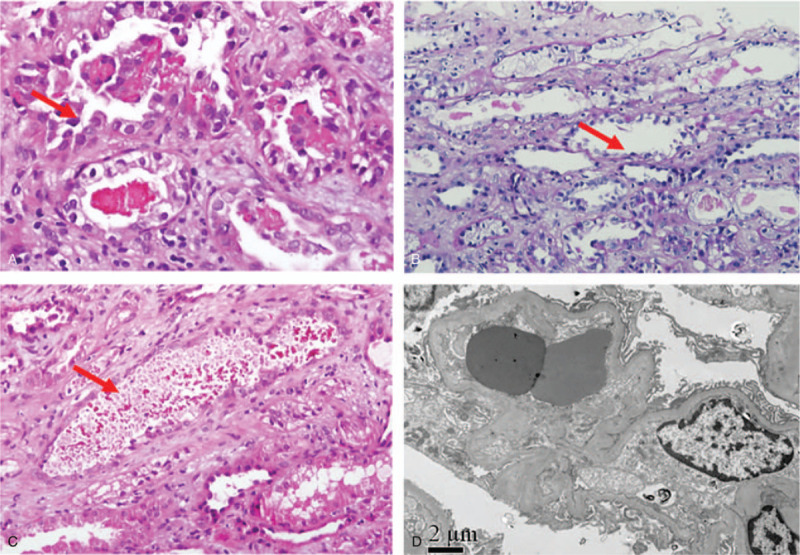
The histological findings of the renal biopsy specimen. (A) The severe vacuolar degeneration of the renal tubular epithelium is shown. (B) The desquamation of the brush border. (C) The granules blocking the renal tubule lumen are shown. (D) The electron microscopic examination revealed the severe vacuolar degeneration of renal tubular epithelial cells, and the segmental foot process fusion. (A: PAS×400; B: PAS×100; C: PAS×400; D: EM×8000.).

At the 21st day after hospitalization, the patient's platelet and hemoglobin recovered to a safe level, and renal biopsy was performed for the pathologic examination. The results revealed severe vacuolar degeneration of the tubular epithelia (Fig. 2A) and disruption of the brush border (Fig. 2B). A large number of high-density brown granules were found to impact the tubular lumen (Fig. 2C), but was compatible with the hemoglobin casts. The immunofluorescence staining for immunoglobulin G (IgG), IgA, IgM, C3, and C1q were all negative. The electron microscopy revealed severe vacuolar degeneration of the renal tubular epithelia and segmental foot process fusion without electron dense deposits (Fig. 2D).

### 2.3 Complement component analysis

After admission, the levels of the complement components were checked. The von Willebrand factor (VWF) activity was 72% (normal: 40--99%). The CFH concentration was 408.9 μg/mL (normal: 247.00–1010.80 μg/mL), and the anti-CFH antibody was negative.

### 2.4 Gene analyses

A mutation was identified in exon 12 of the CFB, which changed a lysine at amino acid position 533 to an arginine (c.1598A>G p.Lys 533Arg). No mutations were identified in the complement sequencing of the coding regions of CFH, CFI, MPC, VWF, diacylglycerol kinase epsilon (DGKE), and C3 genes.

## 3 Discussion

Hemolytic uremic syndrome (HUS) is a rare and serious disorder, and is characterized by intravascular hemolysis, thrombocytopenia, and acute kidney injury.^[4]^ Approximately 30% of HUS patients also develop central nervous system abnormalities and fever.^[6]^ HUS typically follows a diarrhea episode associated with O157:H7 *Escherichia coli* infections.^[5]^ However, 10% of patients with similar presentations do not have diarrhea, and are diagnosed with aHUS.^[4]^ The aHUS portends a poor prognosis.^[6]^ That is, 25% of patients die during the acute phase, and 50% of patients eventually progress to end-stage renal disease (ESRD).^[7]^ It can be challenging to initially make a correct diagnosis of aHUS, but an abrupt onset of hemolytic anemia, thrombocytopenia, and acute kidney injury should prompt the consideration of HUS. For the present case, the evidence of red cell fragmentation (schistocytes and polychromasia) in the peripheral blood smear, elevated LDH, and indirect hyperbilirubinemia aided in the diagnosis of aHUS, and the negative direct Coomb test helped to exclude the possibility of autoimmune hemolytic anemia.

In cases of aHUS, the renal pathology typically presents findings, such as glomerular endothelial damage leading to microthrombi formation within the glomerular capillaries and subsequent endothelial proliferation, thickening of the basement membrane, and the formation of double contours.^[8]^ Surprisingly, for the present case, the renal damage mainly resulted from the hemolytic anemia with hemoglobin cast blocking tubules, which led to acute tubular necrosis. One possible reason can be that the renal biopsy was performed late in the disease course, during which the endothelial damage already recovered, and the endothelial injury was no longer discernible upon pathologic examination. Nonetheless, based on the pathological findings of the present case, the investigators consider that tubular damages in the form of acute tubular necrosis can be another possible presentation in aHUS.

Genetic abnormalities are found in approximately 60% of aHUS patients.^[9,10]^ Most mutations and variants in complement regulatory proteins related to aHUS involve CFH, CFI, MCP, CFHR (complement factor related proteins)1–5, DGKE, and CFB genes.^[2,11–13]^ Among complement-associated HUS, CFB mutations are relatively rare,^[14–17]^ with a frequency of 1% to 2%.^[11]^ The *CFB* gene encodes the factor B protein, which is an important component of the alternate pathway, and its activation provides an active subunit that binds with C3b to form C3 convertase, C3bBb. The catalytic site of C3bBb required for amplifying the alternative pathway is located within the Bb portion of the convertase.^[16]^ In the literature, CFB mutations can enhance the formation of C3 convertase or increase its resistance to inactivation, cause complement 3 overactivation, which causes vascular endothelial injury, the hemolytic anemia, and an episode of aHUS and kidney injury.^[18]^ The investigators reported a pathogenic variant in the *CFB* gene (C.1598A>G), which could cause HUS in 1 literature report.^[19]^ The present report confirms that the mutation can lead to the occurrence of HUS.

Although the present patient had CFB mutations, this patient did not develop aHUS during childhood. The trigger of the patient's aHUS was likely the serious pulmonary infection of the *S. aureus*. The infection can activate the complement system, but a defect in factor B can induce a complement overactivation, and precipitate the episode of aHUS. Consequently, the development of aHUS in the present patient may stem from the interaction between environmental factors and inherent genetic defects.

At present, one of the most effective therapeutic regimens for aHUS is the plasma exchange or infusion for correcting the complement dysregulation.^[20]^ This patient underwent plasma exchange, received antibiotics and hemodialysis, and obtained a favorable outcome. After 15 months of follow-up, the patient remained clinically stable without recurrence.

In conclusion, a rare heterozygous variant c.1598A>G p.Lys 533Arg in the *CFB* gene, which was associated with adult-onset aHUS, was described and successfully treated. More reports of aHUS in patients with CFB gene mutation would likely provide further insights into the pathogenesis of this rare disease, possibly uncovering the underlying mechanisms to aid in the early diagnosis and development of effective therapies.

## Acknowledgment

We thank the patient for his contributions in the research.

## Author contributions

**Conceptualization:** Hao Wu, Li Zhang.

**Data curation:** Hao Wu, Li Zhang.

**Formal analysis:** Hao Wu, Sensen Su.

**Funding acquisition:** Hao Wu, Li Zhang.

**Methodology:** Hao Wu, Lin Li.

**Project administration:** Sensen Su, Li Zhang.

**Software:** Hao Wu, Sensen Su, Lin Li.

**Writing – original draft:** Hao Wu, Sensen Su.

**Writing – review & editing:** Hao Wu, Li Zhang.
